# Synthesis carbonic anhydrase enzyme inhibition and antioxidant activity of novel benzothiazole derivatives incorporating glycine, methionine, alanine, and phenylalanine moieties

**DOI:** 10.1080/14756366.2018.1553040

**Published:** 2018-12-27

**Authors:** Deniz Üzeroğlu Payaz, F. Zehra Küçükbay, Hasan Küçükbay, Andrea Angeli, Claudiu T. Supuran

**Affiliations:** a Department of Chemistry, Faculty of Arts and Sciences, İnönü University, Malatya, Turkey;; b Department of Basic Pharmaceutical Sciences, Faculty of Pharmacy, İnönü University, Malatya, Turkey;; c Dipartimento Neurofarba, Sezione Di Scienze Farmaceutiche E Nutraceutiche e Laboratorio Di Chimica Bioinorganica, Universita` Degli Studi Di Firenze, Florence, Italy

**Keywords:** Benzothiazole; amino acids; carbonic anhydrase; antioxidant; benzotriazole methodology

## Abstract

Thirteen novel benzothiazole derivatives incorporating glycine, methionine, alanine, and phenylalanine were synthesised by facile acylation reactions through benzotriazole or DCC mediated reactions and their structures were identified by ^1^H-NMR, 13C-NMR, and FT-IR spectroscopic techniques and elemental analysis. The carbonic anhydrase (CA, EC 4.2.1.1) inhibitory activity of the new compounds was assessed against four human (h) isoforms, hCA I, hCA II, hCA V, and hCA XIII. Some of the synthesised compounds showed good *in vitro* carbonic anhydrase inhibitory properties, with inhibition constants in the micromolar level. The new amino acid benzothiazole conjugates found to be more effective against hCA V and hCA II inhibition. *In vitro* antioxidant activities of the novel compounds were determined by DPPH method. Most of the synthesised compounds showed moderate to low antioxidant activities compared to the control antioxidant compounds (BHA and α-tocopherol).

## 1. Introduction

Benzothiazole derivatives have been extensively studied in drug chemistry^1^^,^^2^ and they exhibit diverse activities such as antitubercular^3^, antimicrobial^4–6^, antimalarial^7^, anticonvulsant^8^, antioxidant^9^, antidiabetic^10^, antitumor^11^, carbonic anhydrase (CA)^12^, and tryptase inhibitors^13^. Moreover, benzothiazole moieties are also found in fluorescent pH indicators^14^, iminocoumarin based zinc sensors^15^, bioluminogenic agent^16^^,^^17^, vulcanisation process of rubber^18^ and ligands for transition metal catalysts^19^.

On the other hand, CAs are a class of well-studied metalloenzymes that are widely distributed in all living organisms^20^. These enzymes (hCAs) are zinc-containing enzymes that catalyse the reversible hydration of carbon dioxide to bicarbonate and a proton (CO_2_ + H_2_O⇆HCO_3_^−^ + H^+^). Fifteen isoforms of human CA (hCA I–XV) have been isolated, their presence being fundamental for the regulation of many physiological processes^21^^,^^22^.

The interest in finding an effective carbonic anhydrase enzyme inhibitor^12^^,^^23–26^ has been increasing in recent years, especially with the exploring of possible relationships between carbonic anhydrase and cancer^22^^,^^2^,^7–29^. A similar interest is also observed in synthesising effective antioxidants for food products and drugs.

With the hope to obtain an effective carbonic anhydrase enzyme inhibitor having good antioxidant properties, we planned to synthesise new amino acid-benzothiazole conjugates using the benzotriazole methodology and to explore their carbonic anhydrase enzyme inhibition and antioxidant properties.

## 2. Material and methods

### 2.1. Chemistry

The starting materials and reagents used in the reactions were supplied commercially by Acros (Newark, NJ), Aldrich (St. Louis, MO), Fluka (Munich, Germany), or Merck (Kenilworth, NJ). The solvents were dried by standard methods and freshly distilled prior to use. All microwave assisted reactions were carried in a microwave oven system manufactured by Milestone (Milestone Start S Microwave Labstation for Synthesis, Valbrembo, Italy). ^1^H NMR (300.13 MHz) and ^13^C NMR (75.47 MHz) spectra were recorded using a Bruker Avance 300 MHz Ultrashield high performance digital FT NMR spectrometer (Bruker, Billerica, MA). Infrared spectra were recorded as KBr pellets in the range 4000–400 cm*^−^*^1^ on a Perkin Elmer FT-IR spectrophotometer (PerkinElmer, Waltham, MA). Mass spectra were obtained using an Agilent 6460 Series Triple LC/MS instrument (Santa Clara, CA, USA). Elemental analyses were performed with a LECO CHNS-932 elemental analyser (LECO, ST. Joseph, MI). Melting points were recorded using an electrothermal-9200 melting point apparatus (Electrothermal Engineering, Essex, UK) and are uncorrected.

### 2.2. Synthesis of amino acid-benzothiazole derivatives

Benzyl (2-(1H-benzo[d][1,2,3]triazol-1-yl)-2-oxoethyl)carbamate (**I**), tert-butyl (2-(1H-benzo[d][1,2,3]triazol-1-yl)-2-oxoethyl)carbamate (**II**), tert-butyl (1-(1H-benzo[d][1,2,3]triazol-1-yl)-1-oxopropan-2-yl)carbamate (**III**), tert-butyl (1-(1H-benzo[d][1,2,3]triazol-1-yl)-1-oxo-3-phenylpropan-2-yl)carbamate (**IV**), (S)-benzyl (1-(1H-benzo[d][1,2,3]triazol-1-yl)-1-oxo-3-phenylpropan-2-yl)carbamate (**V**), (9H-fluoren-9-yl)methyl (2-(1H-benzo[d][1,2,3]triazol-1-yl)-2-oxoethyl)carbamate (**VI**) and benzyl (1-(1H-benzo[d][1,2,3]triazol-1-yl)-4-(methylthio)-1-oxobutan-2-yl)carbamate (**VII**) were prepared according to the literature procedures^30–33^.

#### 2.2.1. General procedure for the synthesis of benzothiazole conjugates 1–13

A mixture of equivalent amounts of the appropriate N-protected aminoacylbenzotriazole and appropriate benzothiazole derivative was subjected to microwave irradiation (100 W, 70 °C) in anhydrous dichloromethane for 30 min. After the completion of the reaction, all volatiles were removed by rotary evaporator and the obtained crude product was crystallised from ethanol.

#### 2.2.2. Benzyl (3–(6-methylbenzo[d]thiazol-2-yl)-2-oxopropyl)carbamate (1)

*White crystals* (69,88%), mp 213–214 °C. ν_(CO)amide_: 1710 cm^−1^, ν_(CO)carbamate_: 1670 cm^−1^. ^1^H-NMR (DMSO-d_6_) δ: 12.38 (s, 1H, N*H*CO), 7.77 (s, 1H, Ar-*H*), 7.69 (t, 1H, Ar-*H*, *J* = 8.0 Hz), 7.64 (d, 1H, Ar-H, *J* = 8 0.0 Hz), 7.39–7.21 (m, 6H, Ar-*H* + N*H*), 5.07 (s, 2H, C*H_2_*Ph), 4.99 (d, 2H, NH*C*H_2_CO, *J* = 8.0 Hz). 13C-NMR (DMSO-d_6_) δ: 169.1 (NH*C*O), 156.4(CH_2_O*C*O), 146.7 (S*C*HN), 136,9, 133.0, 131.6, 128.3, 127.8, 127.7, 127.4, 127.2, 121.3, 120.2 (Ar-*C*), 65.6 (*C*H_2_Ph), 43.5 (NH*C*H_2_CO), 20.9 (*C*H_3_). Elemental analysis: C_18_H_17_N_3_O_3_S required C, 60.83; H, 4.82; N, 11.82; S, 9.02, found C, 61.04; H, 4.99; N, 11.72; S, 8.89. MS *m/z* for C_18_H_17_N_3_O_3_S [M] ^+^ calcd. 355.1 found 355.9.

#### 2.2.3. Tert-butyl (3–(6-methylbenzo[d]thiazol-2-yl)-2-oxopropyl)carbamate (2)

White crystals (72.6%), mp 212–213 °C. ν_(CO)amide_: 1706 cm^−1^, ν_(CO)carbamate_: 1666 cm^−1^. ^1^H-NMR (DMSO-d_6_) δ: 12.32 (s, 1H, N*H*CO), 7.76 (s, 1H, Ar-*H*), 7.62 (d, 1H, Ar-*H*, *J* = 8.2 Hz), 7.26–7.17 (m, 2H, Ar-*H* + N*H*), 3.89 (d, 2H, NH*CH_2_*CO, *J* = 6.0 Hz), 1.40 (s, 9H, C(C*H*_3_)_3_ . 13C-NMR (DMSO-d_6_) δ: 169.4 (NH*C*O), 156.8(CH_2_O*C*O), 155.9 (S*C*HN), 146,5, 132.9, 131.5, 127.4, 121.3, 120.1 (Ar-*C*), 78.2 (*C*(CH_3_)_3_), 43.2 (NH*C*H_2_CO), 28.1 (C(*C*H_3_)_3_, 20.9 (*C*H_3_). Elemental analysis: C_15_H_19_N_3_O_3_S required C, 56.06; H, 5.96; N, 13.07; S, 9.98, found. C, 55.95; H, 5.80; N, 12.95; S, 9.80, MS *m/z* for C_15_H_19_N_3_O_3_S [M] ^+^ calcd. 321.1 found 321.9.

#### 2.2.4. Tert-butyl (4–(6-methylbenzo[d]thiazol-2-yl)-3-oxobutan-2-yl)carbamate (3)

White crystals (73.0%), mp 207–208 °C. ν_(CO)amide_: 1711 cm^−1^, ν_(CO)carbamate_: 1671 cm^−1^. ^1^H-NMR (DMSO-d_6_) δ: 12.35 (s, 1H, N*H*CO), 7.75 (s, 1H, Ar-*H*), 7.63 (d, 1H, Ar-*H*, *J* = 8.2 Hz), 7.30 (d, 1H, Ar-*H*, *J* = 8.2 Hz), 7.24 (d, 1H, N*H*, *J* = 8.2 Hz), 4.29 (q, 1H, C*H*CH_3_, *J* = 8.0 Hz), 2.41 (s, C*H*_3_), 1,51 (s, 9H, C(C*H*_3_)_3_), 1.27 (d, 3H, CHC*H*_3_, *J* = 8.0 Hz). 13C-NMR (DMSO-d_6_) δ: 172.9 (NH*C*O), 157.0(CH_2_O*C*O), 155.2 (S*C*HN), 146,5, 132.9, 131.6, 127.4, 121.3, 120.1 (Ar-*C*), 78.2 (*C*(CH_3_)_3_), 49.8 (NH*C*HCO), 20.9 (C(*C*H_3_)_3_, 17.4 (*C*H_3_). Elemental analysis: C_16_H_21_N_3_O_3_S required C, 57.29; H, 6.31; N, 12.53; O, 14.31; S, 9.56, found. C, 57.73; H, 6.08; N, 12.53; S, 9.30, MS *m/z* for C_16_H_21_N_3_O_3_S [M] ^+^ calcd. 335.1 found 321.9.

#### 2.2.5. Tert-butyl (4–(6-methylbenzo[d]thiazol-2-yl)-3-oxo-1-phenylbutan-2-yl)carbamate (4)

White crystals (80.4%), mp 167–168 °C. ν_(CO)amide_: 1713 cm^−1^, ν_(CO)carbamate_: 1672 cm^−1^. ^1^H-NMR (DMSO-d_6_) δ: 12.55 (s, 1H, N*H*CO), 7.77 (s, 1H, Ar-*H*), 7.64 (d, 1H, Ar-*H*, *J* = 8.2 Hz), 7.54–6.88 (m, 7H + N*H*), 4.29 (m, 1H, NHC*H*CO), 3.08–3.02 and 2.88–2.80 (m, 2H, C*H*_2_Ph), 2.42 (s, C*H*_3_), 1,32 (s, 9H, C(C*H*_3_)_3_). 13C-NMR (DMSO-d_6_) δ: 171.9 (NH*C*O), 157.0(CH_2_O*C*O), 155.4 (S*C*HN), 146,5, 133.0, 131.6, 129.3, 128.0, 127.4, 126.4, 121.3, 120.1 (Ar-*C*), 78.3 (*C*(CH_3_)_3_), 56.1 (NH*C*HCO), 36.1 (*C*H_2_Ph), 28.1 (*C*H_3_), 21.0 (C(*C*H_3_)_3_. Elemental analysis: C_22_H_25_N_3_O_3_S required C, 63.98; H, 6.13; N, 10.28; S, 7.77, found. C, 64.21; H, 6.12; N, 10.21; S, 7.79, MS *m/z* for C_22_H_25_N_3_O_3_S [M] ^+^ calcd. 411.2 found 411.9.

#### 2.2.6. Benzyl (3–(6-ethoxybenzo[d]thiazol-2-yl)-2-oxopropyl)carbamate (5)

White crystals (67.27%), mp 214–215 °C. ν_(CO)amide_: 1712 cm^−1^, ν_(CO)carbamate_: 1673 cm^−1^. ^1^H-NMR (DMSO-d_6_) δ: 7.84–7.10 (m, 8H, Ar-*H* + N*H*), 6.92 (dd, 1H, Ar-*H*, *J* = 2.4 Hz, 1H), 5.06 (s, 2H, C*H*_2_Ph), 4.04 (q, 2H, C*H*_2_CH_3_, *J* = 6.9 Hz), 3.88 (d, 2H, C*H*_2_CO, *J* = 5.8 Hz), 1.34 (t, 3H, CH_2_C*H*_3_, *J* = 6.9 Hz). 13C-NMR (DMSO-d_6_) δ: 170.6 (NH*C*O), 156.4(CH_2_O*C*O), 155.7, 154.5 (S*C*HN), 143.3, 137,1, 133.1, 128.3, 127.8, 127.7, 120.2, 114.3, 105.3 (Ar-*C*), 65.4 (*C*H_2_Ph), 63.5 (O*C*H_2_CH_3_) , 44.5 (NH*C*H_2_CO), 14.7 (CH_2_*C*H_3_). Elemental analysis: C_19_H_19_N_3_O_3_S required C, 59.21; H, 4.97; N, 10.90; S, 8.32, found C, 59.14; H, 4.95; N, 10.41; S, 7.92. MS *m/z* for C_19_H_19_N_3_O_3_S [M] ^+^ calcd. 385.1 found 385.9.

#### 2.2.7. Benzyl (4–(6-ethoxybenzo[d]thiazol-2-yl)-3-oxo-1-phenylbutan-2-yl)carbamate (6)

White crystals (71%), mp 160–161 °C. ν_(CO)amide_: 1713 cm^−1^, ν_(CO)carbamate_: 1675 cm^−1^. ^1^H-NMR (DMSO-d_6_) δ: 12.57 (s, 1H, N*H*), 7.86 (d, 1H, N*H*, *J* = 8.1 Hz), 7.74–6.66 (m, 13H, Ar-*H*), 4.97 (s, 2H, C*H_2_*Ph), 4.74–4.40 (m, 1H, C*H*), 4.75 (q, 2H, C*H*_2_CH_3_, *J* = 8.0 Hz), 3.13–3.07 and 2.90–2.82 (m, 2H, CHC*H*_2_), 1,36 (t, 3H, CH_2_C*H*_3_, *J* = 8.0 Hz). 13C-NMR (DMSO-d_6_) δ: 171.5 (NH*C*O), 156.0 (CH_2_O*C*O), 155.7 (S*C*HN), 155.4, 142.5, 137,4, 136.8, 132.8, 129.3, 128.3, 128.1, 127.8, 127.6, 121.1, 115.2, 105.4 (Ar-*C*), 65.4 (*C*H_2_Ph), 63.6 (NH*C*HPh), 56.4 (O*C*H_2_CH_3_) , 36.9 (CH*C*H_2_Ph), 14.7 (CH_2_*C*H_3_). Elemental analysis: C_26_H_25_N_3_O_4_S required C, 65.67; H, 5.30; N, 8.84; S, 6.74, found C, 65.62; H, 5.25; N, 8.74; S, 6.72. MS *m/z* for C_26_H_25_N_3_O_4_S [M] ^+^ calcd. 475.2 found 476.0.

#### 2.2.8. Tert-butyl (3–(6-ethoxybenzo[d]thiazol-2-yl)-2-oxopropyl)carbamate (7)

White crystals (86%), mp 171–172 °C. ν_(CO)amide_: 1710 cm^−1^, ν_(CO)carbamate_: 1670 cm^−1^. ^1^H-NMR (DMSO-d_6_) δ: 12.25 (s, 1H, N*H*), 7.63 (d, 1H, Ar-*H*, *J* = 12.0 Hz), 7.55 (d, 1H, Ar-*H*, *J* = 4.0 Hz), 7.19 (t, 1H, NH, *J* = 8.0 Hz) 7.01 (dd, 1H, Ar-*H*, *J* = 8.8 and 2.6 Hz), 4.06 (q, 2H, C*H_2_*CO, *J* = 6.9 Hz), 3.88 (d, 2H, C*H*_2_CO, *J* = 8.0 Hz), 1,41 (s, 9H, C(C*H*_3_)_3_), 1.35 (t, 3H,CH_2_C*H*_3_, *J* = 6.9 Hz). 13C-NMR (DMSO-d_6_) δ: 169.2 (NH*C*O), 155.9(CH_2_O*C*O), 155.6 (S*C*HN), 155.3, 142.5, 132,7, 121.1, 115.2, 105.4 (Ar-*C*), 78.2 (*C*H_2_Ph), 63.6 (O*C*H_2_CH_3_) , 43.1 (NH*C*H_2_CO), 28.1 (C(*C*H_3_)_3_), 14.6 (CH_2_*C*H_3_). Elemental analysis: C_16_H_21_N_3_O_4_S required C, 54.68; H, 6.02; N, 11.96; S, 9.12, found C, 54.77; H, 5.98; N, 11.88; S, 9.02. MS *m/z* for C_19_H_19_N_3_O_3_S [M] ^+^ calcd. 351.1 found 352.0.

#### 2.2.9. Benzyl (3–(6-(methylsulfonyl)benzo[d]thiazol-2-yl)-2-oxopropyl)carbamate (8)

White crystals (63%), mp 178–179 °C. ν_(CO)amide_: 1714 cm^−1^, ν_(CO)carbamate_: 1677 cm^−1^. ^1^H-NMR (DMSO-d_6_) δ: 13.03 (s, 1H, N*H*), 8.67 (s, 1H, Ar-*H*), 7.96 (d, 3H, N*H*, *J* = 4.0 Hz + Ar-*H*), 7.40–7.27 (m, 10H, Ar-*H*), 4.98 (s, 1H, C*H_2_*Ph), 4.74–4.40 (m, 1H, C*H*), 3.27 (s, 3H, CH_3_), 3.16–3.10 and 2.99–2.62 (m, 2H, CHC*H*_2_). ^13^C-NMR (DMSO-d_6_) δ: 172.4 (NH*C*O), 161.8 (*C*-SO_2_Me), 156.4 (CH_2_O*C*O), 152.0 (S*C*HN), 137.2, 136.7, 135,5, 132.0, 129.3, 128.3, 128.1, 127.9, 127.6, 126.6, 124.9, 122.1, 120.9 (Ar-*C*), 65.3 (*C*H_2_Ph), 56.6 (*C*HCH_2_Ph), 44.0 (*C*H_3_), 36.7 (CH*C*H_2_Ph). Elemental analysis: C_25_H_23_N_3_O_5_S_2_ required C, 58.92; H, 4.55; N, 8.25; S, 12.58, found C, 58.95; H, 4.56; N, 8.26; S, 12.61. MS *m/z* for C_25_H_23_N_3_O_5_S_2_ [M]^+^ calcd. 509.1 found 510.1.

#### 2.2.10. (9H-Fluoren-9-yl)methyl (2-((6-methylbenzo[d]thiazol-2-yl)amino)-2-oxoethyl)carbamate (9)

White crystals (81%), mp 220–221 °C. ν_(CO)amide_: 1709 cm^−1^, ν_(CO)carbamate_: 1671 cm^−1^. ^1^H-NMR (DMSO-d_6_) δ: 12.42 (s, 1H, N*H*CO), 7.91 (d, 2H, Ar-*H*), 7.82–7.70 (m, 4H, Ar-*H* + N*H*), 7.44 (t, 2H, Ar-*H*, *J* = 7.6 Hz), 7.36 (t, 2H, Ar-H, *J* = 7.6 Hz), 7.30–7.14 (m, 2H, Ar-*H*) , 4.50–4.15 (m, 3H, C*H* + C*H*_2_O), 4.99 (d, 2H, NH*CH_2_*CO, *J* = 6.0 Hz), 2.58 (s, 3H, C*H*_3_). 13C-NMR (DMSO-d_6_) δ: 169.7 (NH*C*O), 157.4(CH_2_O*C*O), 157.1 (S*C*HN), 148,1, 144.3, 141.2, 130.3, 128.1, 127.6, 127.1, 125.7, 124.0, 120.6, 119.6, 110.2 (Ar-*C*), 66.3 (*C*H_2_OCO), 47.1 (*C*H), 43.9 (NH*C*H_2_CO), 18.4 (*C*H_3_). Elemental analysis: C_25_H_21_N_3_O_3_S required C, 67.70; H, 4.77; N, 9.47; S, 7.33, found C, 67.54; H, 4.65; N, 9.32; S, 7.19. MS *m/z* for C_25_H_21_N_3_O_3_S [M] ^+^ calcd. 443.1 found 443.7.

#### 2.2.11. (9H-Fluoren-9-yl)methyl(2-((6-(methylsulfonyl)benzo[d]thiazol-2-yl)amino)-2-oxoethyl)carbamate (10)

White crystals (70%), mp 230–211 °C. ν_(CO)amide_: 1711 cm^−1^, ν_(CO)carbamate_: 1674 cm^−1^. ^1^H-NMR (DMSO-d_6_) δ: 12.77 (bs, 1H, N*H*CO), 8.66 (s, 1H, Ar-*H*), 7.99–7.87 (m, 4H, Ar-*H*) , 7.81 (t, 1H, N-*H*, *J* = 6 Hz), 7.74 (d, 2H, Ar-H, *J* = 7.6 Hz), 7.74 (t, 2H, Ar-*H*, *J* = 7.2 Hz), 7.36 (t, 2H, Ar-*H*, *J* = 7.2 Hz), 4.41–4.16 (m, 3H, C*H* + C*H*_2_O), 4.07 (d, 2H, NH*CH_2_*CO, *J* = 6.0 Hz), 3.26 (s, 3H, C*H*_3_). 13C-NMR (DMSO-d_6_) δ: 170.5 (NH*C*O), 162.3 (*C*-SO_2_Me), 157.1 (CH_2_O*C*O), 152,6 (S*C*HN), 144.3, 141.2, 135.9, 132.5, 128.1, 127.6, 125.7, 125.3, 122.6, 121.3, 120.6 (Ar-*C*), 66.3 (*C*H_2_OCO), 47.1 (*C*H), 44.5 (NH*C*H_2_CO), 44.1 (*C*H_3_). Elemental analysis: C_25_H_21_N_3_O_5_S_2_ required C, 59.16; H, 4.17; N, 8.28; S, 12.63, found C, 58.90; H, 4.34; N, 7.97; S, 12.10. MS *m/z* for C_25_H_21_N_3_O_5_S_2_ [M] ^+^ calcd. 507.1 found 507.9.

#### 2.2.12. (9H-Fluoren-9-yl)methyl (2-((4-methylbenzo[d]thiazol-2-yl)amino)-2-oxoethyl)carbamate (11)

White crystals (76%), mp 218–219 °C. ν_(CO)amide_: 1713 cm^−1^, ν_(CO)carbamate_: 1672 cm^−1^. ^1^H-NMR (DMSO-d_6_) δ: 12.35 (s, 1H, N*H*CO), 7.91 (d, 2H, Ar-*H*, *J* = 7.5 Hz), 7.83–7.70 (m, 4H, Ar-*H* + N*H*), 7.63 (d, 1H, Ar-*H*, *J* = 8.4 Hz), 7.25 (d, 2H, Ar-H, *J* = 7.6 Hz), 7.43 (t, 2H, Ar-*H*, *J* = 7.4 Hz), 7.34 (t, 2H, Ar-*H*, *J* = 7.4 Hz), 7.25 (d, 1H, Ar-*H*, *J* = 8.4 Hz), 4.39–4.18 (m, 3H, C*H* + C*H*_2_O), 3.99 (d, 2H, NH*CH_2_*CO, *J* = 6.0 Hz), 2.41 (s, 3H, C*H*_3_). 13C-NMR (DMSO-d_6_) δ: 169.6 (NH*C*O), 157.4 (S*C*HN), 157.1 (CH_2_O*C*O), 144,3, 141.2, 133.5, 128.1, 127.6, 125.7, 121.9, 121.8, 120.7, 120.6, 120.5, 110.2 (Ar-*C*), 66.3 (*C*H_2_OCO), 47.1 (*C*H), 44.0 (NH*C*H_2_CO), 21.4 (*C*H_3_). Elemental analysis: C_25_H_21_N_3_O_3_S required C, 67.70; H, 4.77; N, 9.47; S, 7.23, found C, 67.54; H, 4.65; N, 9.32; S, 7.19. MS *m/z* for C_25_H_21_N_3_O_3_S [M] ^+^ calcd. 443.1 found 443.9.

#### 2.2.13. Benzyl (1-((4–(6-methylbenzo[d]thiazol-2-yl)phenyl)amino)-1-oxo-3-phenylpropan-2-yl)carbamate (12)

White crystals (70,62%), mp 227–228 °C. ν_(CO)amide_: 1711 cm^−1^, ν_(CO)carbamate_: 1673 cm^−1^. ^1^H-NMR (DMSO-d_6_) δ: 10.47 (s, 1H, N*H*), 8.25–7.52 (m, 7H, Ar-*H* + N*H*), 7.45–6.80 (m, 11H, Ar-*H*), 4.99 (s, 2H, C*H_2_*Ph), 4.56–4.35 (m, 1H, C*H*), 3.15–2.99 (m, 1H, C*H*HPh), 2.98– 2.80 (m, 1H, C*H*HPh), 2.45 (s, 3H, CH_3_). 13C-NMR (DMSO-d_6_) δ: 171.0 (NH*C*O), 165.8 (S*C*HN), 156.0 (CH_2_O*C*O), 151.8, 141.44, 137.70, 136.88, 135.00, 134.41, 129.22, 128.28, 128.08, 128.00, 127.83, 127.72, 127.56, 126.39, 122.13, 121.75, 119.50 (Ar-*C*), 65.4 (*C*H_2_Ph), 57.1 (*C*HCH_2_Ph), 37.3 (CH*C*H_2_Ph) , 21.1 (*C*H_3_). Elemental analysis: C_31_H_27_N_3_O_3_S required C, 71.38; H, 5.22; N, 8.06; S, 6.15, found C, 71.22; H, 5.21; N, 8.08; S, 6.17. MS *m/z* for C_31_H_27_N_3_O_3_S [M] ^+^ calcd. 521.2 found 521.9.

#### 2.2.14. Benzyl (1-((4–(6-methylbenzo[d]thiazol-2-yl)phenyl)amino)-4-(methylthio)-1-oxobutan-2-yl)carbamate (13)

Beige crystals (70,85%), mp 200–201 °C. ν_(CO)amide_: 1713 cm^−1^, ν_(CO)carbamate_: 1676 cm^−1^. ^1^H-NMR (DMSO-d_6_) δ: 10.42 (s, 1H, N*H*), 8.04 (d, 2H, Ar-*H*), 7.98–7.86 (m, 2H, Ar-*H* +N*H*), 7.82 (d, 2H, Ar-*H*, *J* = 8.7 Hz), 7.75 (d, 1H, Ar-*H*, *J* = 7.7 Hz), 7.49–6.91 (m, 6H, Ar-*H*), 5.06 (s, 2H, C*H_2_*Ph), 4.37–4.20 (m, 1H, C*H*), 2.64–2.48 (m, 5H, C*H*_2_ + C*H*_3_), 2.45 (s, 3H, C*H*_3_), 2.00–1.65 (m, 1H, C*H_2_S*). 13C-NMR (DMSO-d_6_) δ: 171.1 (NH*C*O), 165.8 (S*C*HN), 156.1 (CH_2_O*C*O), 151.8, 141.5, 136.9, 135.0, 134.4, 128.3, 127.8, 122.1, 121.7, 119.5(Ar-*C*), 65.5 (*C*H_2_Ph), 54.8 (*C*HCH_2_CH_2_S), 31.3 (*C*H_2_CH_2_S) , 229.7 (*C*H_3_), 21.01 (S*C*H_3_), 4.6 (*C*H_2_SCH_3_). Elemental analysis: C_27_H_27_N_3_O_3_S_2_ required C, 64.13; H, 5.38; N, 8.31; S, 12.68, found C, 64.17; H, 5.28; N, 8.43; S, 12.71. MS *m/z* for C_27_H_27_N_3_O_3_S^2^ [M] ^+^ calcd. 505.2 found 505.9.

### 2.3. CA inhibition

An applied photophysics stopped-flow instrument has been used for assaying the CA catalysed CO_2_ hydration activity by using method of Khalifah^34^. Phenol red (at a concentration of 0.2 mM) has been used as an indicator, working at the absorbance maximum of 557 nm, with 20 mM HEPES (pH 7.5) as buffer, and 20 mM Na_2_SO_4_ (for maintaining constant the ionic strength), following the initial rates of the CA-catalysed CO_2_ hydration reaction for a period of 10–100 s. The CO_2_ concentrations ranged from 1.7 to 17 mM for the determination of the kinetic parameters and inhibition constants. For each inhibitor at least six traces of the initial 5–10% of the reaction have been used for determining the initial velocity. The uncatalysed rates were determined in the same manner and subtracted from the total observed rates. Stock solutions of inhibitor (0.1 mM) were prepared in distilled–deionised water and dilutions up to 0.01 nM were done thereafter with the assay buffer. Inhibitor and enzyme solutions were preincubated together for 15 min at room temperature prior to assay, in order to allow the formation of the E–I complex. The inhibition constants were obtained by non-linear least-square methods using PRISM (www.graphpad.com), and non-linear least squares methods, values representing the mean of at least three different determinations, as described earlier by us^35–39^.

### 2.4. Antioxidant testing

#### 2.4.1. DPPH radical scavenging activity

Antioxidant activity was determined based on the ability of the antioxidants to act as radical scavengers towards the stable free radical, 1,1-diphenyl-2-picrylhydrazyl (DPPH). As detailed by Yang et al.^40^, 1 ml of antioxidant solution (solubilised in ethanol) was added to 3 ml of a 0.1 mM ethanolic solution of DPPH. After 30 min at ambient temperature in darkness, absorbance readings were taken at 517 nm. Inhibition (%) was calculated using the equation

[1–(As−Ao)/Ab]×100

where As is the absorbance reading for samples containing antioxidant, Ao is the absorbance of the antioxidant in pure methanol and Ab corresponded to the absorbance of the DPPH solution.

## 3. Results and discussion

Benzothiazole is an important scaffold for drug development, because it exhibits a broad spectrum of pharmacological activities^1^^,^^2^. In this study, 13 new amino acid–benzothiazole derivatives were successfully synthesised and their human carbonic anhydrase enzyme inhibition and antioxidant capacities were determined by using the stopped flow methodology and DPPH method.

Among the carbonic anhydrase (CA, EC 4.2.1.1) enzymes (four human (h) isoforms, hCA I, hCA II, hCA V, and hCA XIII) the new amino acid benzothiazole conjugates showed more effective inhibitory activity against hCA V and hCA II than hCA I and hCA XII. *In vitro* antioxidant activities of the novel compounds were determined by DPPH method. Most of the synthesised compounds showed moderate to low antioxidant activities compared to the control antioxidant compounds (BHA and α-tocopherol).

### 3.1. Synthesis and characterisation of the new amino acid–benzothiazole derivatives

**Scheme 1.**
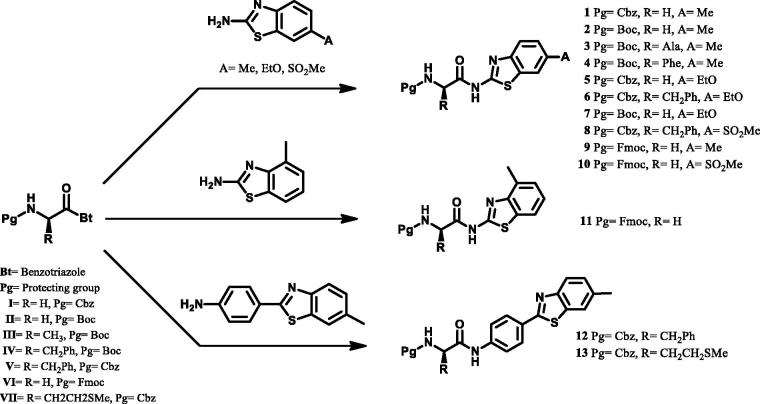
Synthetic pathways of amino acid-benzothiazole derivatives (**1**–**13**).

The syntheses of novel amino acid–benzothiazole derivatives reported in this study are depicted in Scheme 1. Because benzotriazole acts as easy leaving group, we chose benzotriazole-mediated methodology to synthesise the desired amino acid benzothiazole conjugates. Compounds **1–13** were prepared through a facile benzotriazole mediated acylation reaction in one step (Scheme 1) at 70 °C under microwave irradiation for 30 min in dry dichloromethane with good or high yields. To learn the effect of the protection group, we used several protection groups such as Cbz, Boc, or Fmoc. All the compounds were fully characterised by ^1^H, 13C NMR, MS, and FTIR (ATR) spectroscopy and elemental analyses. All spectral data were in agreement with the proposed structures. The characteristic NH resonances of the benzothiazole part of the amino acid–benzothiazole conjugates **1**–**13** were observed at 10.42–13.03 ppm region as singlet peak in the ^1^H NMR spectrum. The carbamate NH resonances of compounds **3**, **6**, **7**, **8**, and **10** were observed at 7.24, 7.86, 7.19, 7.96, and 7.8 ppm as triplet, respectively, whereas for compounds, **1**, **2**, **4**, **5**, **9**, **11**, **12,** and **13** were observed in the aromatic region together with aromatic protons. Both NH protons were confirmed by D_2_O exchange. The singlet that peaks around 5.00 ppm for compounds **1**, **5**, **6**, **8**, **12,** and **13** was assigned to the CH_2_ protons for benzyloxycarbonyl protected group whereas the upfield singlet that signals around 1.40 ppm was assigned to the *tert*-butyl protons of Boc-protected group for compounds, **2**, **3**, **4,** and **7**. Carbonyl resonances of the amide carbonyls and carbamate carbonyl were observed around 171 and 156 ppm, respectively. All other aliphatic and aromatic protons and carbons were observed in the expected regions. The molecular ion peaks were observed for all proposed structures of novel compounds in the mass spectra. The IR spectra of amino acid–benzothiazole conjugates, **1–13,** showed characteristic amide carbonyl peaks around between 1706 and 1719 cm^−1^, whereas the carbamate carbonyl peaks around between 1666 and 1677 cm^−1^. All other spectral data were in accordance with the assumed structures.

### 3.2. Carbonic anhydrase inhibition

All the synthetised amino acid-benzothiazole conjugates have been evaluated by means of a stopped flow CO_2_ hydrase assay^34^ to test their inhibitory potency against four human (h) CA isoforms (hCA I, hCA II, hCA V, and hCA XIII). Inhibition data are reported in Table 1, along with those referred to acetazolamide (AAZ), used as standard sulphonamide inhibitor. In order to evaluate the effect of substitution at both amino and benzothiazole parts of the amino acid–benzothiazole conjugates several new compounds have been prepared from the reaction of the corresponding N-protected amino acid and appropriate benzothiazole moieties. The following structure–activity relationship (SAR) has been delineated:

**Table 1.** Inhibition data of hCA I, hCA II, hCA V, and hCA XIII with compounds **1–13** and the standard sulfonamide inhibitor acetazolamide (**AAZ**) by a stopped flow CO_2_ hydrase assay.

K_I_ (µM)^a^				
Comp	hCA I	hCAII	hCA V	hCA XIII
**1**	4.3	32.1	4.3	94.6
**2**	>100	74.8	65.5	>100
**3**	90.5	50.4	86.9	>100
**4**	>100	>100	>100	>100
**5**	>100	82.3	>100	>100
**6**	>100	>100	>100	>100
**7**	>100	>100	60.0	>100
**8**	89.9	>100	9.0	>100
**9**	71.9	>100	7.3	>100
**10**	7.1	>100	2.9	>100
**11**	>100	88.1	74.5	>100
**12**	>100	37.0	>100	>100
**13**	>100	39.1	41.2	84.9
**AAZ**	0.25	0.012	0.063	0.017

^a^Mean from three different assays, by a stopped flow technique (errors were in the range of ±5–10% of the reported values).

- Compounds **1**, **3**, **8**–**10** showed Ki values in the low micromolar levels ranging from 4.3 to 90.5 µM (Table 1). Among these compounds, Me and SO_2_Me substituents at 6 position of the benzothiazole incorporating glycine, alanine and phenylalanine made positive contribution to the inhibition activity.
- Compounds **1**–**3**, **5,** and **11**–**13** showed considerable inhibition against CA II with Ki values in the low micromolar levels, ranging from 32.1 to 88.1 µM (Table 1).
- As seen in Table 1, nearly all compounds displayed potent inhibition against CA V with Ki values in the low micromolar levels, ranging from 2.9 to 86.9 µM, except compounds **4**–**6** and **12**.
- Among the compounds, only compounds **1** and **13** showed some inhibition against the tumour associated isoform CA XII with Ki values with 94.6 and 84.9 µM, respectively.

### 3.3. Antioxidant testing

#### 3.3.1. DPPH radical scavenging activity

The antioxidant activity of the compounds was determined based on the ability of the antioxidants to act as radical scavengers towards the stable free radical, 1,1-diphenyl-2-picrylhydrazyl (DPPH)^40^.

The antioxidant results of the new compounds were given in Table 2. Among the tested compounds seen in Scheme 1, the compounds bearing ethoxy group at 6 position of the benzothiazole ring was found to be the most effective antioxidant at 62.5 and 125 μg/ml concentrations with 32 and 38%. The compounds bearing methylsulfonyl at 6 position of the benzothiazole ring was found to be next effective antioxidant at 62.5 and 125 μg/ml concentrations with 22 and 23%. With respect to the amino acid part of the compounds, glycine and phenylalanine derivatives seemed to be more active for the antioxidant activities.

**Table 2.** Antioxidant activities of the synthesised amino acid-benzothiazole derivatives.

	Antioxidant activity, %				
Comp. no	12.5 μg/ml	25 μg/ml	37.5 μg/ml	62.5 μg/ml	125 μg/ml
**1**	0	0	0	11.4	12.5
**2**	0	0	3.9	4.6	6.1
**3**	3.3	3.4	5.3	7.5	7.7
**4**	0	5.7	5.8	9.6	10,4
**5**	12.0	15.0	25.0	32.0	38.0
**6**	11.0	14.0	23.0	32.0	37.0
**7**	13.0	15.0	22.5	24.8	28.3
**8**	0	2.1	6.6	19.2	20.7
**9**	4	4.6	5.3	5.8	6.4
**10**	0	0	22.5	23.1	24.5
**11**	5	6.7	6.9	7.1	8.2
**12**	0	3	3.4	5.8	6.1
**13**	5	6.7	6.1	7.1	8.2
α-Toc.	62.9	63.4	68.4	72.8	74.0
BHA	61.1	63.0	67.5	71.0	72.4

## 4. Conclusions

In this study, 13 new amino acid-sulfathiazole conjugates were synthesised, and their carbonic anhydrase inhibitory properties were determined against human carbonic anhydrase hCA I, hCA II, hCA V, and hCA XII. The new amino acid-benzothiazole conjugates showed considerable inhibition against, hCA V and hCA II with Ki values in the micromolar levels, ranging from 2.9 to 88.1 µM. Among the compounds, compound **1** showed potent enzyme inhibition against all carbonic anhydrase enzymes studied in this work with micromolar levels. *In vitro* antioxidant activities of the novel compounds were determined by DPPH method. On the other hand, most of the synthesised compounds showed moderate to low antioxidant activities compared to the control antioxidant compounds (BHA and α-tocopherol).
